# A case report of parotid gland epithelioid hemangioendothelioma

**DOI:** 10.3389/fsurg.2024.1367059

**Published:** 2024-04-22

**Authors:** Ke Wang, Jianhong Hu, Jiazhu Wen, Shuxia Zhou, Linfeng Ye, Chun Fang, Jiacheng Guan, Xiao Luo

**Affiliations:** ^1^ Department of Radiology, The Second People’s Hospital of Quzhou, Quzhou, China; ^2^ Department of Pathology, The Second People’s Hospital of Quzhou, Quzhou, China; ^3^ Department of Radiology, The Second Affiliated Hospital of Zhejiang University School of Medicine, Hangzhou, China

**Keywords:** epithelioid hemangioendothelioma, MRI, parotid gland, imaging diagnosis, surgical treatment

## Abstract

Epithelioid hemangioendothelioma (EHE) is a rare low-grade malignant vascular tumor. It mainly occurs in the liver, lungs, bones, and other parts of the body. Reports of epithelioid hemangioendothelioma in the parotid gland are rare in both domestic and international literature. Here, we present a case report of a parotid gland epithelioid hemangioendothelioma, including its complete clinical course and imaging findings, to improve the diagnosis and treatment of this unusual disease.

**Case presentation:**

The patient, a 75-year-old female, presented with a swelling around the right ear for 2 months and pain for 20 days. Enhanced MRI of the parotid gland revealed a well-defined, round mass with homogeneous signal intensity. The mass showed low signal intensity on T1-weighted imaging, high signal intensity on T2-weighted imaging, nodular low signal intensity within, significant high signal intensity on DWI sequence, low signal intensity on ADC sequence, and heterogeneous enhancement in the arterial phase after intravenous injection of Gd-DTPA. Nodular non-enhancing low signal intensity was observed internally, and slight clearance was seen in the venous phase. The initial diagnosis before surgery was a benign lesion, but after histopathological and immunohistochemical examination, it was confirmed as epithelioid hemangioendothelioma.

**Intervention:**

Complete tumor resection was performed.

**Results:**

The patient experienced a favorable recovery, with meticulous follow-up conducted for up to 1 year revealing no signs of recurrence or metastasis. Continued patient surveillance is ongoing to substantiate and validate the long-term efficacy of the treatment.

**Conclusion:**

Due to the extreme rarity of parotid gland epithelioid hemangioendothelioma, it often leads to a high misdiagnosis rate. The most common misdiagnosis is salivary gland lymphoma, followed by epithelioid hemangiosarcoma. When the lesion is multifocal, fusiform, with internal necrosis, and shows punctate low signal intensity on T2-weighted imaging, significant enhancement in the arterial phase, particularly with more pronounced peripheral enhancement, and persistent enhancement in the venous and delayed phases, epithelioid hemangioendothelioma should be considered. However, the current clinical diagnosis of epithelioid hemangioendothelioma still primarily relies on immunohistochemical methods.

## 1 Introduction

Epithelioid hemangioendothelioma (EHE) is a rare low-grade malignant vascular tumor, first described and named by Weiss and Enzinger in 1982 (1). It was classified as an intermediate-grade vascular tumor in the World Health Organization (WHO) classification of tumors in 1994. In the updated classification in 2013, it was reclassified as a malignant vascular tumor in soft tissues (1).

The tumor is composed of nest-like arrangements of epithelioid or histiocytoid cells with a distinctive glassy or chondromyxoid stromal component (2). The etiology and pathogenesis of EHE remain poorly understood; however, it may be associated with factors such as trauma, radiation injury, hormonal therapy, long-term oral contraceptive use, among others (3). Notably, epithelioid hemangioendothelioma can manifest at any age, but it is typically diagnosed between the ages of 20–30 years, with a slightly higher prevalence in women compared to men (4). EHE has a slow clinical course and exhibits biological behavior between that of benign vascular tumors and malignant angiosarcomas. It typically presents as multiple lesions, although solitary cases can occur. Initially, it manifests as localized masses, but later it can invade surrounding tissues and cause corresponding symptoms. Metastasis can occur, and the local recurrence rate after surgery is approximately 20%–30%, with a mortality rate of 15%, highlighting the complexity of managing this rare condition (1). Furthermore, we have provided key epidemiological data, such as an incidence of 0.038 per 100,000 per year and a prevalence of <1 in 1,000,000 (5), In the literature, EHE mainly occurs in the liver, lungs, bones, and other organs, and reports of EHE in the parotid gland are extremely rare (6). This report presents a case of parotid gland epithelioid hemangioendothelioma with typical clinical features, analyzes the clinical presentation, imaging characteristics, and surgical treatment plan in detail, and provides an overview of the disease.

## 2 Clinical data

The patient was a 75-year-old female who presented with a complaint of “swelling around the right ear for 2 months, with pain for 20 days.” The patient reported noticing a grape-sized swelling in front of her right ear while washing her face 2 months ago. The swelling was tender but was left untreated. Recently, the swelling had significantly increased in size, reaching approximately the size of a quail egg. The consistency of the swelling was soft. Initially, the patient sought treatment at a local health clinic, taking traditional Chinese medicine for a week with no improvement. She then visited the Otolaryngology Department of our hospital and received intravenous treatment for 2 days, which showed limited effectiveness. Therefore, she was admitted to our hospital for further treatment.

**Figure 1:**
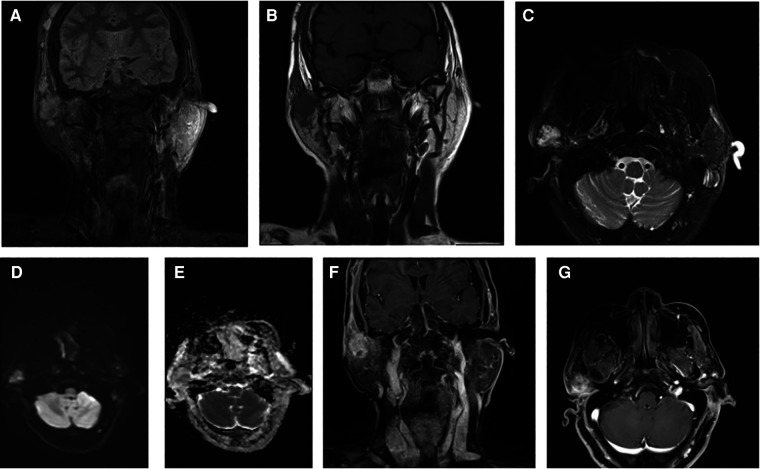
Combined magnetic resonance imaging (MRI) findings: (**A**) coronal T2-weighted imaging (T2WI) fat-suppressed sequence showing a high signal intensity mass. (**B**) Coronal T1-weighted imaging (T1WI) sequence displaying a low signal intensity mass. (**C**) Transverse T2WI fat-suppressed sequence revealing a high signal intensity mass. (**D**) Diffusion-weighted imaging (DWI) sequence indicating a signal in the mass. (**E**) Apparent diffusion coefficient (ADC) sequence showing a low signal intensity in the tumor. (**F**,**G**) Contrast-enhanced scans depicting the mass with evident heterogeneous enhancement.

**Figure 2:**
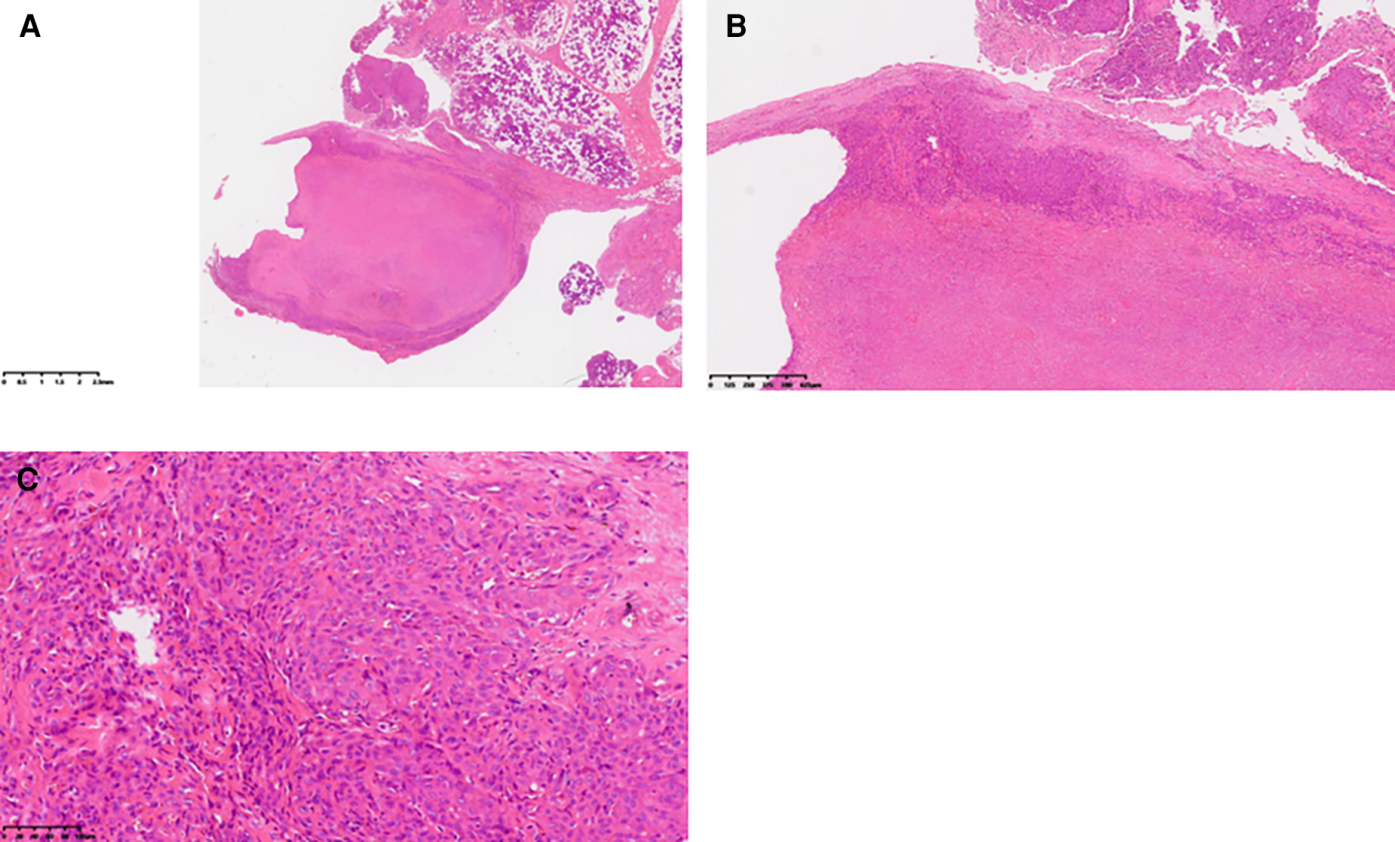
Pathological findings: (**A**) gross view of the tumor under low magnification. (**B**) Under medium magnification, epithelioid tumor cells were distributed in patches, with cytoplasm rich in acid. Intracytoplasmic vacuoles were visible in some tumor cells, and obvious interstitial vitreous changes were noted. (**C**) At high magnification, tumor cells exhibited rich cytoplasm, eosinophilic appearance, vesicular nucleus, inconspicuous nucleoli, no observed nuclear division image, and arrangement in small nests.

**Figure 3:**
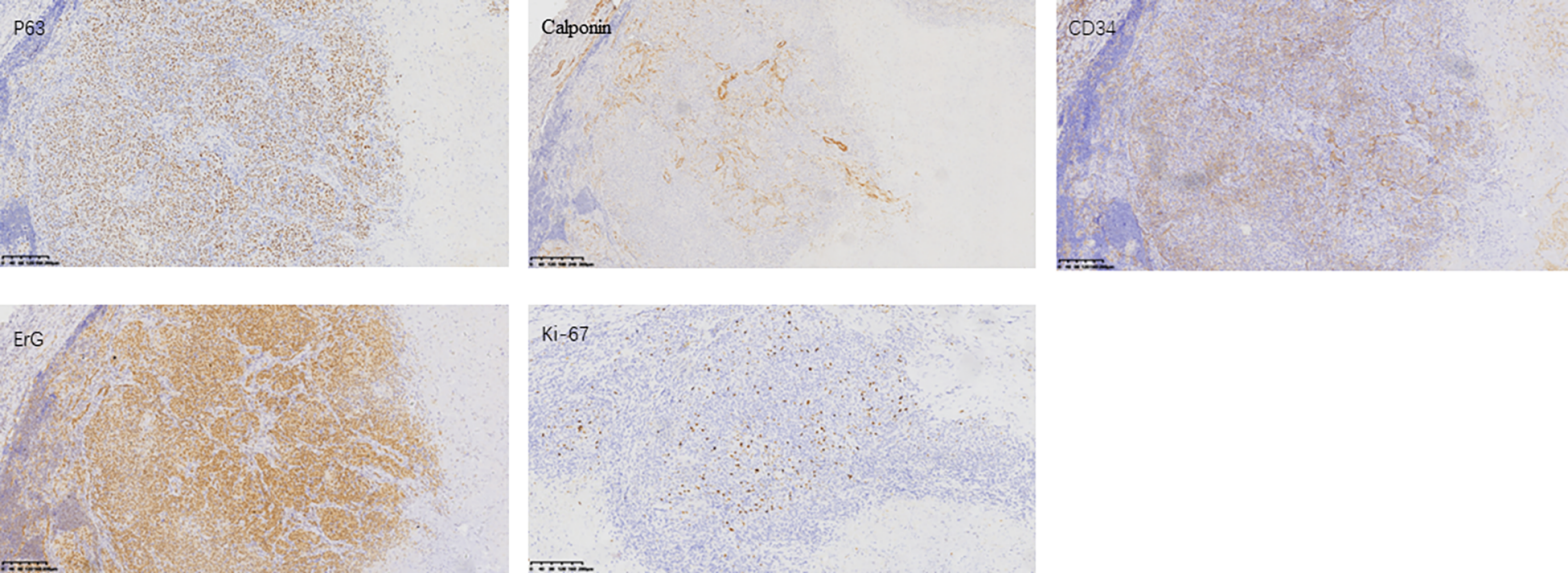
Immunohistochemistry pictures: formalin-fixed paraffin-embedded (FFPE) sections from the tumor were assessed by IHC. Representative images of P63, Calponin, CD34, ErG, and Ki-67 expression staining are shown.

Upon admission, a specialized examination revealed symmetrical bilateral facial features. A palpable swelling of approximately 2 cm × 2 cm was found in front of the right ear. The swelling had clear borders, a soft texture, a slightly elevated surface, and was significantly tender. The patient had normal facial movements and could close her eyes and open her mouth with a three-finger width of mouth opening. No crepitus was detected in the preauricular area. A parotid gland MRI showed a mass in the right parotid gland with T1 hypointense and T2 hyperintense signals, measuring approximately 29 × 25 × 28 mm. The lesion exhibited significant hyperintensity on the DWI sequence and hypointensity on the ADC sequence. After intravenous injection of GD-DTPA contrast agent, the arterial phase of the enhancement scan showed significant heterogeneous enhancement, while the venous phase showed slight clearance. A cystic T2 hypointense signal was observed internally, and no significant enhancement was seen in the cystic area after contrast enhancement. The diagnosis was a benign lesion. (Figure 1). With the consent of the patient and her family, surgical excision was performed for treatment. The surgical pathology report indicated a low-grade malignant spindle and epithelioid cell tumor in the right parotid gland (Figure 2). Immunohistochemical staining results showed negative CK, CK5/6, S100, and CD117; positive P63, Calponin, ErG, CD34; and approximately 5% Ki-67 expression (hot spot area). HHV8 staining was negative (Figure 3).

## 3 Surgical procedure

The patient was placed in a supine position. After confirming the information, endotracheal intubation was performed under general anesthesia. Routine disinfection, draping, and towel placement were carried out with the head turned to the left side. An arc-shaped incision was made in front of the right ear, and the skin was sequentially dissected layer by layer. The skin flap was elevated from the surface of the fascia, exposing the superficial lobe of the parotid gland. Blunt dissection was performed from the preauricular region to the cheek, separating the superficial lobe of the parotid gland. The tumor was observed in the deep portion of the superficial lobe, with unclear boundaries. Careful and meticulous dissection of the tumor and surrounding parotid tissue was performed. The tumor and a portion of the parotid gland were completely removed. Adjacent flaps were used for layer-by-layer approximation and suturing of muscle tissue, subcutaneous tissue, and skin. A negative pressure drainage bottle was placed. At the end of the surgery, the operative field was in good condition. Intraoperatively, facial nerve decompression was performed, and intraoperative bleeding was approximately 20 ml. Routine pathological examination was performed on the excised mass. The patient was safely transferred back to the recovery room. Intraoperative frozen section examination suggested the possibility of a myoepithelial-like tumor with necrosis, but myoepithelial carcinoma could not be ruled out.

## 4 Discussion

EHE is a rare tumor, and its occurrence in the parotid gland is extremely rare. There have been only a few reported cases in domestic and international literature, mostly presented as isolated case reports. The majority of patients with EHE do not experience significant discomfort or symptoms and are often incidentally discovered. Clinically, it commonly presents as painless nodules in superficial or deep tissues. Due to the tumor's vascular origin, there may be associated edema or hemorrhagic manifestations, and some patients may experience pain, swelling, or discomfort in the affected area. Due to the low incidence of this disease and the presence of multiple lesions on imaging, misdiagnosis can easily occur (7). Currently, the main approach to definitive diagnosis of this disease is through surgical excision to obtain tissue specimens for pathological and immunohistochemical examination.

Imaging examinations of EHE initially show focal soft tissue masses, either solitary or multiple. In later stages, scattered soft tissue density shadows may appear, often in a punctate distribution pattern. On contrast-enhanced scans, irregular or ring-enhancement can be observed. MRI findings typically include low signal intensity on T1-weighted images, heterogeneous high signal intensity on T2-weighted images, and the presence of punctate areas of low signal intensity within the lesion. Enhanced scans show abundant blood flow, with lesion enhancement consistent with vascular enhancement, which is characteristic of EHE. CT scans may reveal invasive bone destruction or well-defined expansile lytic lesions. Areas of hemorrhage may also be seen, appearing as areas of high density (8, 9).

Pathological histological features of EHE: Grossly, EHE presents as a solid or cystic mass. Tumor cells originate from blood vessels and can cause vascular involvement, resulting in dilation and compression of surrounding tissues. Histologically, EHE is composed of epithelioid cells arranged in nests, cords, or strands, demonstrating endothelial cell differentiation. The tumor is typically composed of epithelioid cells with evident vacuoles and red blood cells, along with a glassy eosinophilic cytoplasm, low mitotic activity, and mild nuclear atypia (10). Immunohistochemical staining shows strong positivity for at least one endothelial marker among CD31, CD34, and FVIII antigen. However, SMA, S100, Desmin, PCK, HMB45, and SPA are negative (4). In this case, the tumor was located deep within the right superficial lobe of the parotid gland with indistinct borders. Histologically, epithelioid cells were observed distributed within a glassy matrix, and the surrounding area showed infiltrating inflammatory cells. Additionally, small areas of atypical vascular-derived cells were present, and immunohistochemistry demonstrated CD34 (+) and S100 (−), indicating that the tumor was consistent with EHE. In classical epithelioid hemangioendothelioma, the characteristic WWTR1-CAMTA1 fusion gene is present, while cases with significant angiogenesis have been identified with the YAP1-TFE3 fusion gene (5). When occurring in the parotid gland, this tumor needs to be differentiated from adenolymphoma and epithelioid angiosarcoma. Deyrup et al. classified epithelioid hemangioendothelioma of soft tissue as a high-risk tumor if it has more than 3 mitotic figures per 50 high-power fields or a size larger than 3 cm. These features are not considered low risk. They reported a 5-year disease-specific survival rate of 59% and a metastasis rate of 32% for the high-risk group (1, 11).

In terms of treatment, due to EHE being a rare malignant tumor, there is currently no standardized treatment regimen, and the efficacy is uncertain. Overall, treatment should be individualized based on each patient’s specific circumstances. Surgical resection is the preferred option in clinical practice, and for solitary and localized lesions, complete removal should include the tumor, its capsule, and surrounding normal tissue. For patients who have undergone surgery or have no surgical opportunities, chemotherapy and radiation therapy should be considered. Currently, there is no universally effective chemotherapy regimen. EHE is moderately sensitive to radiation therapy as a soft tissue tumor, but the results are not satisfactory. However, there are still numerous reports in the literature suggesting that radiation therapy for EHE in locations such as the brain, mediastinum, and skeleton can reduce recurrence and alleviate symptoms (5). This work has several limitations. The first one is that there are no post-operative imaging data available for the patient. The patient did not undergo regular imaging examinations in our hospital after surgery, and we can only assess the patient’s current condition through telephone follow-up. The second limitation is that the patient did not undergo relevant genetic testing.

## Data Availability

The original contributions presented in the study are included in the article/Supplementary Material, further inquiries can be directed to the corresponding author.
